# Supplementary material for: IMA peptides regulate root nodulation and nitrogen homeostasis by providing iron according to internal nitrogen status
Source: Nat Commun. 2024 Jan 29;15:733. doi: 10.1038/s41467-024-44865-4 (PMC10825120; doi:10.1038/s41467-024-44865-4)
Supplement: Supplementary file 1 — Supplementary Figures [file 41467_2024_44865_MOESM1_ESM.pdf]

Supplementary Figures

IMA peptides regulate root nodulation and nitrogen homeostasis by providing iron upon internal nitrogen status

Ito et al.

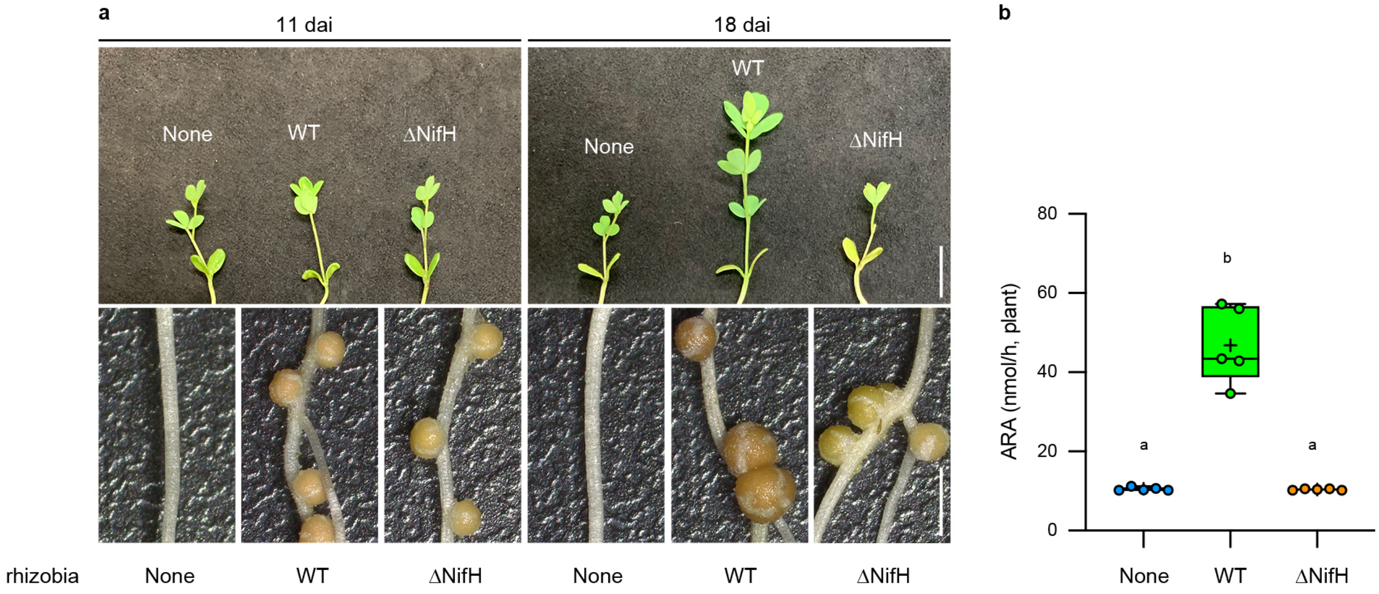

Supplementary Fig. 1 Shoot and nodulation phenotypes of *L. japonicus* WT plants inoculated with ΔNifH rhizobia. **a.** Shoot and nodulation phenotype of *L. japonicus* WT plants inoculated with WT or ΔNifH rhizobia 11 and 18 dai. **b.** ARA of WT plants grown in respective conditions 11 dai (n = 5 plants). Scale bars, 1 cm (upper), 2 mm (lower). Centerlines in the boxplots show the medians, and box limits indicate the 25th and 75th percentile. The whiskers go down to the smallest value and up to the largest. Scatterplots show individual biological replicates as dots. Different letters indicate statistically significant differences ($P < 0.0001$, one-way ANOVA followed by multiple comparisons).

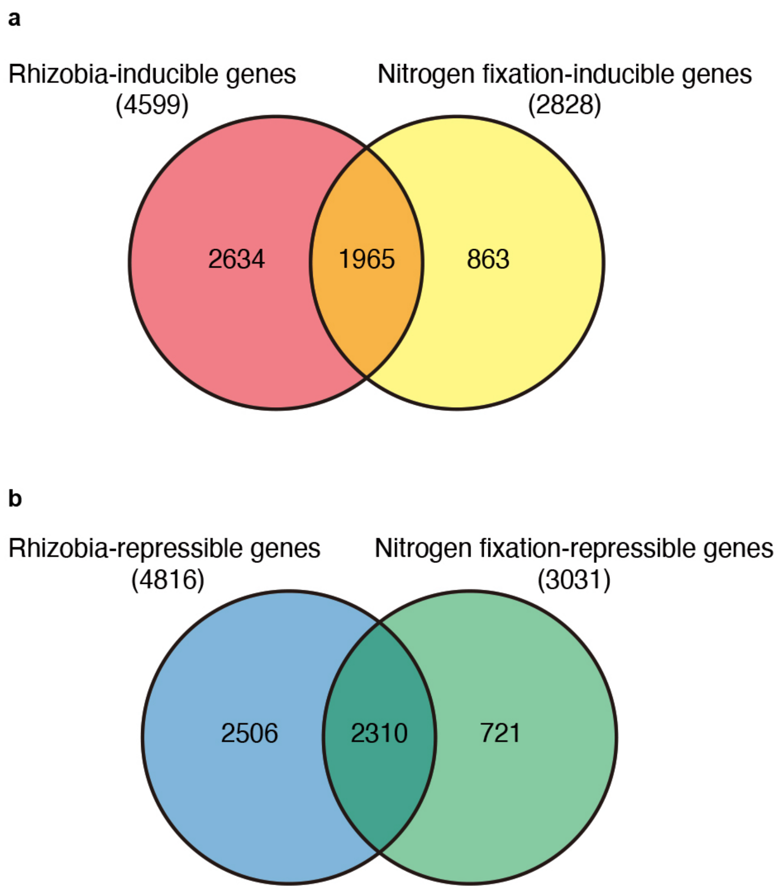

Supplementary Fig. 2 The number of DEG in transcriptome analysis.

a. Venn diagram showing the number of genes with rhizobia-inducible (4,599 genes) and nitrogen fixation-inducible (2,828 genes) pattern in the WT shoot 11 dai. **b.** Venn diagram showing the number of genes with rhizobia-repressible (4,816 genes) and nitrogen fixation-repressible (3,031 genes) pattern in the WT shoot 11 dai. Genes upregulated （$\log_2$FC > 1 and FDR < 0.05） or downregulated ($\log_2$FC < -1 and FDR < 0.05) between non-inoculated and WT rhizobia-inoculated conditions or WT and ΔNifH rhizobia-inoculated conditions were extracted based on select criteria. For the details of the data, see Supplementary Dataset 1.

a

b

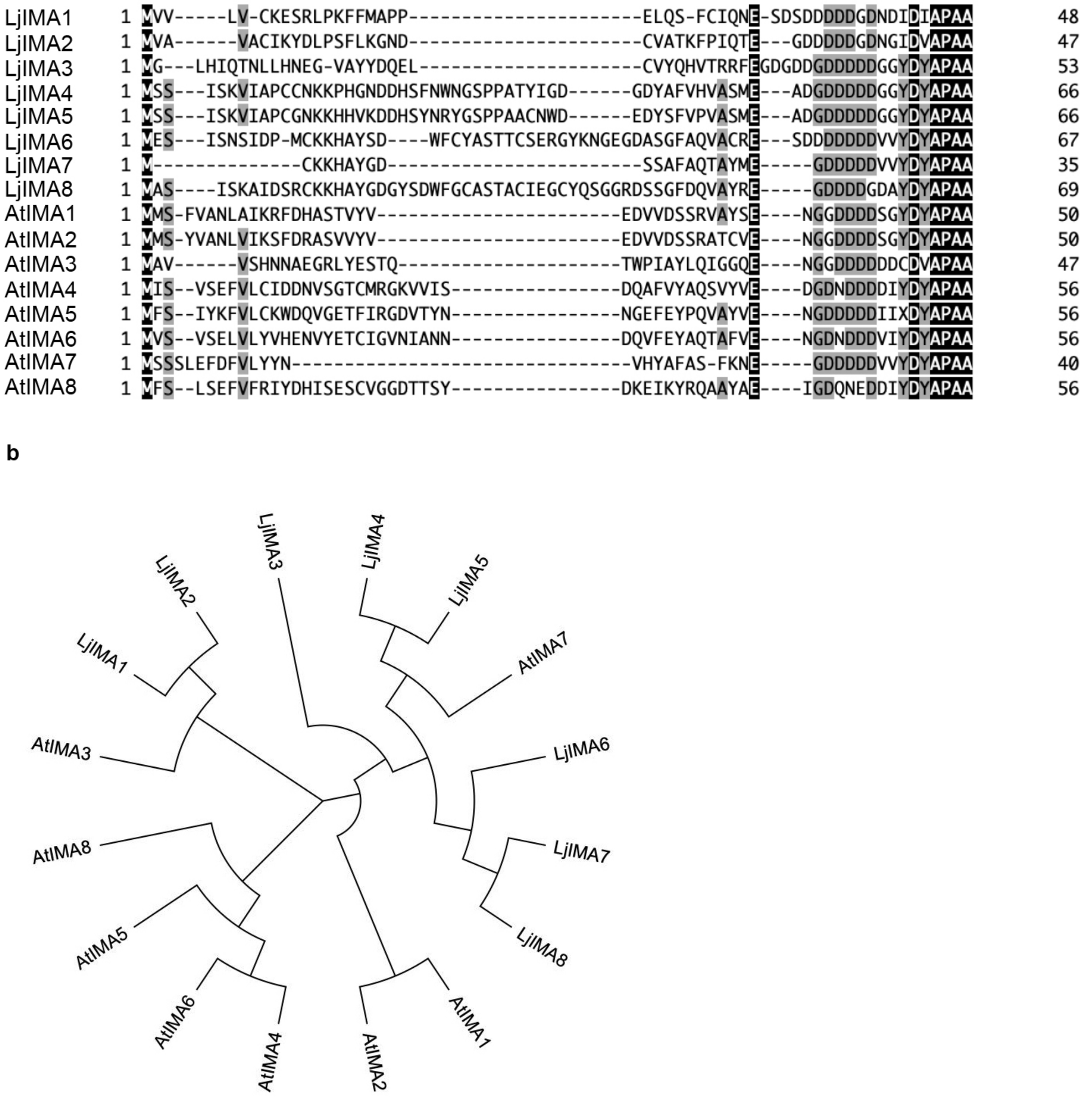

Supplementary Fig. 3 Alignment and phylogenetic tree of Lj/AtIMAs.

Full-length amino acids sequence of LjIMA1 (LC770146), LjIMA2 (LC770147), LjIMA3 (LC770148), LjIMA4 (LC770149), LjIMA5 (LC770150), LjIMA6 (LC770151), LjIMA7 (LC770152), LjIMA8 (LC770153), AtIMA1 (AT1G47400), AtIMA2 (AT1G47395), AtIMA3 (AT2G30766), AtIMA4 (AT1G07367), AtIMA5 (AT1G09505), AtIMA6 (AT1G07373), AtIMA7 (AT2G00920) and AtIMA8 (AT1G47401) are used to create the alignment and phylogenetic tree.

LjIMA1

TTCTGTTCTTTGCTGCAATAATGGTGGTCTTGGTTTGCAAAGAATCTCGTCTTCCCAAGTTTTTCATGGCTCCACCTGAATTACAAAGCT
TTTGCATCCAGAATGAGAGTGATAGTGACGACGATGATGATGGTGATAATGATATTGATATAGCGCCAGCAGCATAG

LjIMA2

TCGTTGCCATCTGTGTCTACACAATATGGTGGCTGTTGCTTGCATCAAATATGACCTGCCTTCGTTTCTTAAGGGCAATGACTGCGTAG
CAACTAAATTTCCCATTCAAACTGAAGGTGATGACGATGATGATGGTGACAATGGCATCGATGTGGCACCAGCAGCATAG

LjIMA1* in *Ljima1* and *Ljima1/2

TTCTGTTCTTTGCTGCAATAA--56 bp deletion --
GAATTACAAAGCTTTTGCATCCAGAATGAGAGTGATAGTGACGACGATGATGATGGTGATAATGATATTGATATAGCGCCAGCAGCATAG

LjIMA2* in *Ljima2

TCGTTGCCATCTGTGTCTACACAATAT---42 bp deletion--
TAAGGGCAATGACTGCGTAGCAACTAAATTTCCCATTCAAACTGAAGGTGATGACGATGATGATGGTGACAATGGCATCGATGTGGCA
CCAGCAGCATAG

LjIMA2* in *Ljima1/2

TCGTTGCCATCTGTGTCTA---31 bp deletion + 1 bp insertion--
ATGACCTGCCTTCGTTTCTTAAGGGCAATGACTGCGTAGCAACTAAATTTCCCATTCAAACTGAAGGTGATGACGATGATGATGGTGAC
AATGGCATCGATGTGGCACCAGCAGCATAG

AtIMA1

ATGATGTCTTTTGTCGCAAACTTGGCCATCAAGAGATTTGACCATGCTTCCACCGTGTATGTTGAAGATGTGGTAGATAGTTCTCGAGT
GGCATATAGTGAGAATGGTGGTGATGACGATGACAGTGGCTATGATTATGCTCCTGCTGCGTGA

AtIMA2

ATGATGTCTTACGTTGCTAACTTGGTCATCAAGAGTTTTGACCGTGCTTCCGTGGTGTATGTTGAAGATGTGGTGGATAGCTCTCGAGC
GACATGTGTTGAGAATGGTGGTGATGACGATGACAGTGGCTATGATTATGCTCCTGCTGCGTGA

AtIMA3

ATGGCAGTGGTGAGTCACAACAACGCAGAAGGCAGGCTATACGAATCAACTCAGACTTGGCCAATTGCTTACTTACAAATTGGTGGCC
AAGAGAACGGAGGAGACGATGACGACGATGACTGTGACGTTGCACCGGCGGCTTGA

AtIMA1* in *Atima1/2/3

ATGATGTCTTTTGTCGCAAACTTGGCCATCAAGAGATTTGACCATGCTTCCACCG----------------------------1 bp insertion---------------------
TGTATGTTGAAGATGTGGTAGATAGTTCTCGAGTGGCATATAGTGAGAATGGTGGTGATGACGATGACAGTGGCTATGATTATGCTCCT
GCTGCGTGA

AtIMA2* in *Atima1/2/3

ATGATGTCTTACGTTGCTA--1 bp insertion--
ACTTGGTCATCAAGAGTTTTGACCGTGCTTCCGTGGTGTATGTTGAAGATGTGGTGGATAGCTCTCGAGCGACATGTGTTGAGAATGG
TGGTGATGACGATGACAGTGGCTATGATTATGCTCCTGCTGCGTGA

AtIMA3* in *Atima3* and *Atima1/2/3

ATGGCAGTGGTGAGTCACAACAACGCAGAA---1 bp insertion---
GGCAGGCTATACGAATCAACTCAGACTTGGCCAATTGCTTACTTACAAATTGGTGGCCAAGAGAACGGAGGAGACGATGACGACGAT
GACTGTGACGTTGCACCGGCGGCTTGA

Supplementary Fig. 4 Locations of mutations in the knockout plants created by the CRISPR-Cas9 system in this study.

Underlines indicate the coding sequence. The positions of gRNAs are highlighted in blue. In *Ljima1*, *Ljima2*, and *Ljima1/2*, the initiation codon of *LjIMA1* and/or *LjIMA2* are deleted, causing a complete loss of function of the gene. In *Atima3* and *Atima1/2/3*, 1 bp insertion in *AtIMA1*, *AtIMA2*, and/or *AtIMA3* causes the frame-shifted mutation.

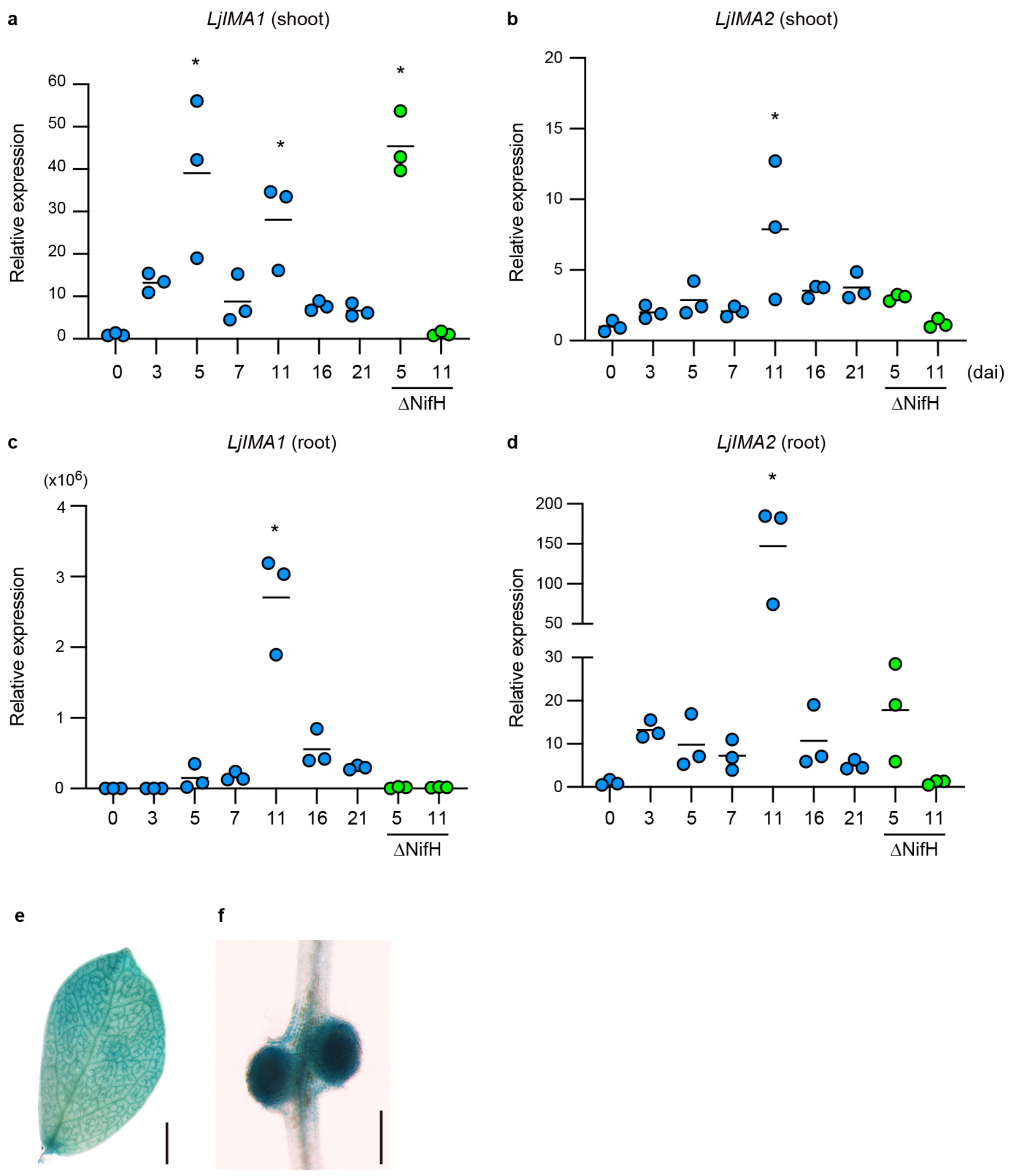

Supplementary Fig. 5 Spatiotemporal *LjIMA1/2* expression patterns.

a-d. RT-qPCR analysis of *LjIMA1/2* expression in the WT shoots and roots during root nodule symbiosis (n = 3 independent pools of shoots or roots derived from three plants). RT-qPCR data were normalized to 0 dai conditions. The expression of *LjUBQ* was used as the reference. **e.f.** Spatial expression of *LjIMA1*. GUS activities in a leave (**e**) or 7 dai roots (**f**) of $LjIMA1_{pro}$*:GUS* plants are shown. Three independent experiments were conducted to obtain similar results. Scale bars, 2 mm in **e.** and 500 µm in **f.** Scatterplots show individual biological replicates as dots. In **b-d.**, bars indicate mean values. In **b-d.**, asterisks indicate a statistically significant difference compared with 0 dai ($P < 0.01$, one-way ANOVA followed by multiple comparisons).

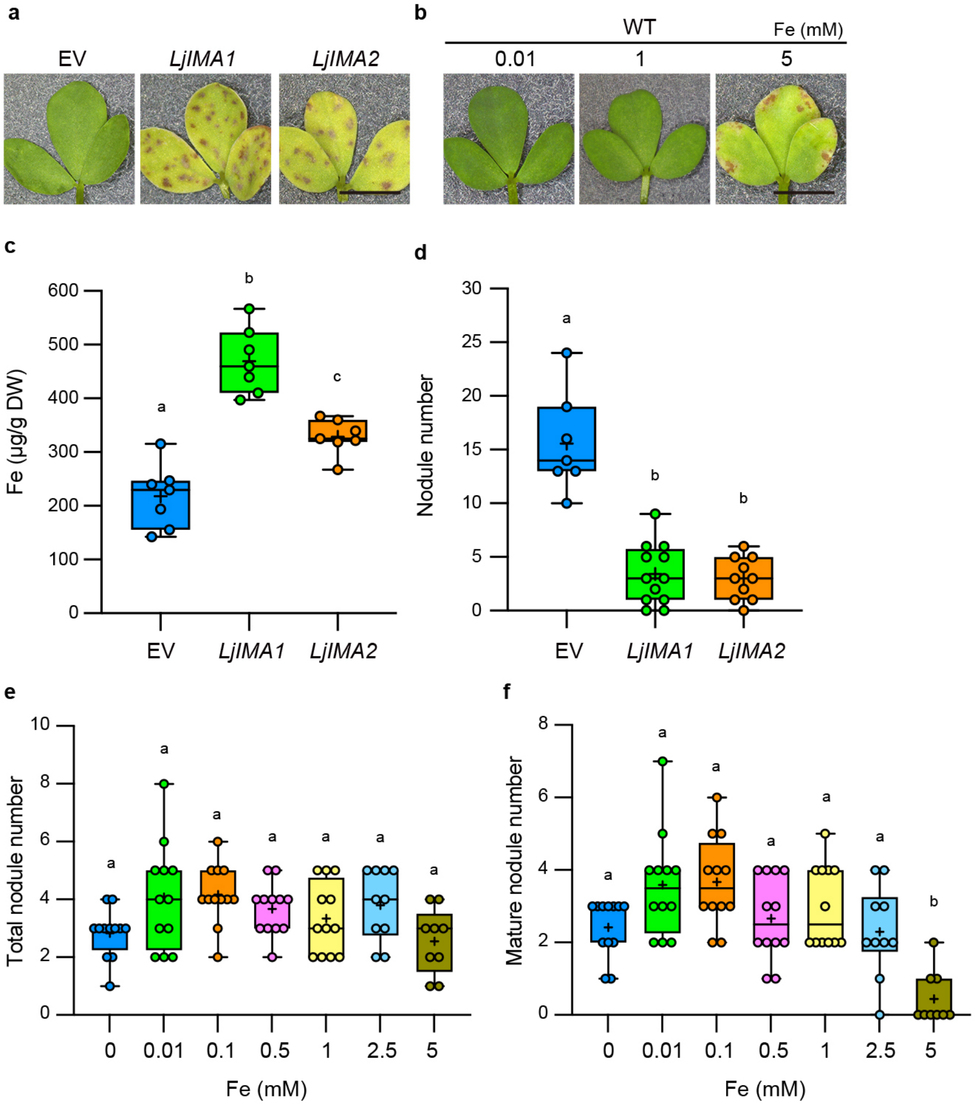

Supplementary Fig. 6 Effects of *LjIMA1/2* overexpression and supply of excessive Fe on leaf phenotype and nodulation in *L. japonicus*.

a. Leaf phenotypes of the WT plants with transgenic hairy roots carrying EV, *LjUBQ*$_{pro}$*:LjIMA1* or *LjUBQ*$_{pro}$*:LjIMA2* constructs. **b.** Leaf phenotypes of the WT plants grown with different Fe concentrations. **c.** Fe amounts of the WT plants with transgenic hairy roots carrying EV, *LjUBQ*$_{pro}$*:LjIMA1* or *LjUBQ*$_{pro}$*:LjIMA2* in non-symbiotic conditions (n = 7 independent pools of hairy roots from three plants). **d.** Total nodule number of the WT plants with transgenic hairy roots carrying EV (n = 7 plants), *LjUBQ*$_{pro}$*:LjIMA1* (n = 12 plants) or *LjUBQ*$_{pro}$*:LjIMA2* (n = 10 plants) constructs 21 dai. In **a.c.d.**, plants were grown in vermiculite with Broughton and Dilworth solution containing 10 µM Fe. **E.f.** Total and mature nodule number of WT plants treated with different Fe concentrations 14 dai (n = 9-12 plants). Fe (III)-EDTA was used for the Fe source. Scale bars, 3 mm. Centerlines in the boxplots show the medians, and box limits indicate the 25th and 75th percentile. The whiskers go down to the smallest value and up to the largest. Scatterplots show individual biological replicates as dots. In **c-f.**, different letters indicate statistically significant differences (P < 0.05, one-way ANOVA followed by multiple comparisons).

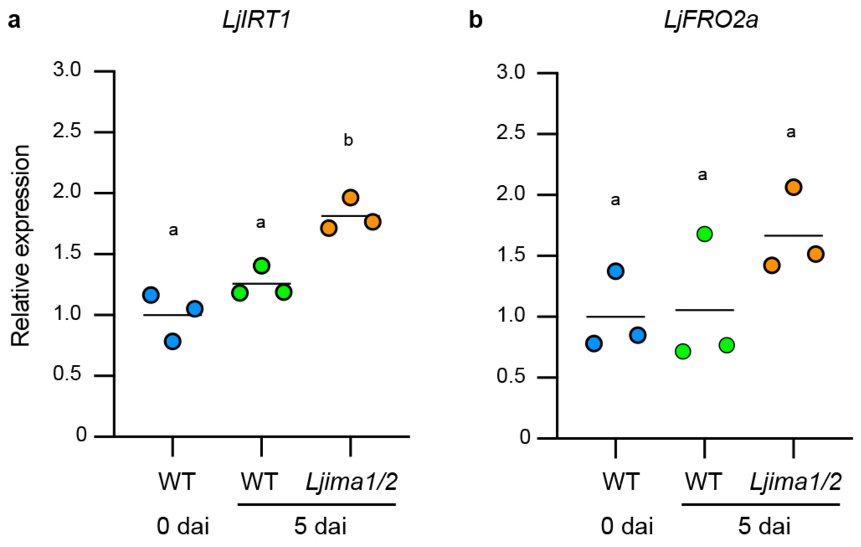

Supplementary Fig. 7 *LjIRT1* and *LjFRO2* expressions 5 dai.

a.b. RT-qPCR analysis of *LjIRT1* and *LjFRO2a* expression in WT and *Ljima1/2*. Non-inoculated (0 dai) or 5 dai roots were collected (n = 3 independent pools of roots derived from three plants). RT-qPCR data were normalized to WT 0 dai conditions. The expression of *LjUBQ* was used as the reference. Scatterplots show individual biological replicates as dots. Bars indicate mean values. Different letters indicate statistically significant differences ($P < 0.05$, one-way ANOVA followed by multiple comparisons).

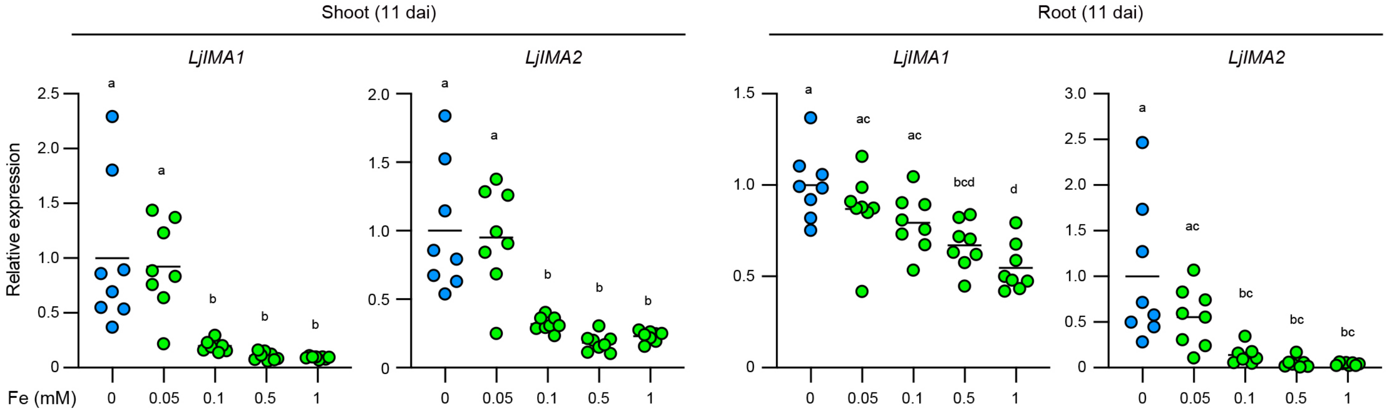

Supplementary Fig. 8 Effects of different concentrations of Fe on *LjIMA1/2* expression at 11dai.

RT-qPCR analysis of *LjIMA1/2* expression in WT. Shoots or roots of 11 dai plants with different concentrations of Fe were collected (n = 8 independent pools of shoot or roots derived from three plants). RT-qPCR data were normalized to –Fe conditions. The expression of *LjUBQ* was used as the reference. Fe (III)-EDTA was used for the Fe source. Scatterplots show individual biological replicates as dots. Bars indicate mean values. Different letters indicate statistically significant differences ($P < 0.05$, two-way ANOVA followed by multiple comparisons).

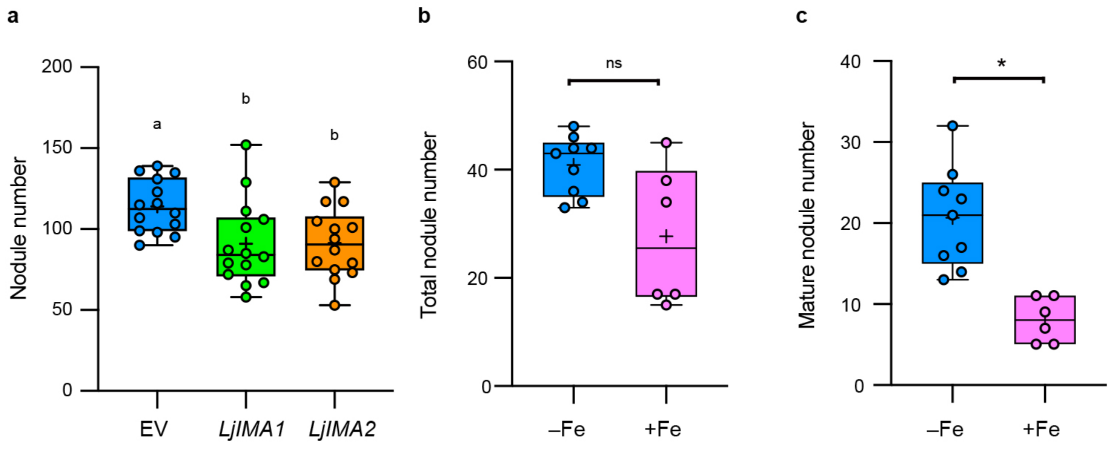

Supplementary Fig. 9 Potential relationship between LjIMA1/2 and AON.

a. Total nodule number of *har1* plants with transgenic hairy roots carrying EV, *LjUBQ*$_{pro}$*:LjIMA1* or *LjUBQ*$_{pro}$*:LjIMA2* constructs 21 dai (n = 14 plants). **b.c.** Total and mature nodule number of WT (n = 9 plants) and *har1* (n = 6 plants) plants treated with 1 mM Fe 14 dai. Fe (III)-EDTA was used for the Fe source. Centerlines in the boxplots show the medians, and box limits indicate the 25th and 75th percentile. The whiskers go down to the smallest value and up to the largest. Scatterplots show individual biological replicates as dots. Different letters indicate statistically significant differences ($P < 0.05$, one-way ANOVA followed by multiple comparisons). Asterisks indicate a statistically significant difference ($P < 0.05$, by a two-sided Welch's *t* test). ns means not significant.

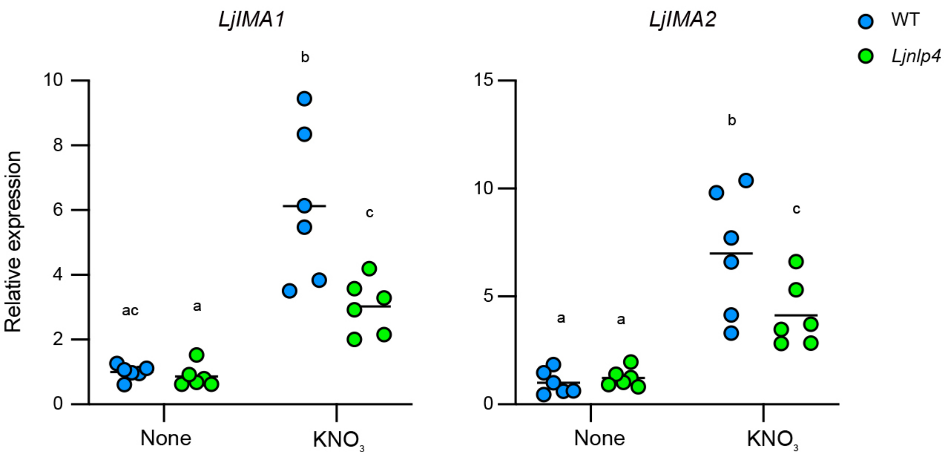

Supplementary Fig. 10 Effects of nitrate treatment on *LjIMA1/2* expression in the root in non-symbiotic conditions. RT-qPCR analysis of *LjIMA1/2* expression in the WT and *Ljnlp4* plants roots 6 h after treatment of 5 mM KNO_3 in the absence of rhizobia (n = 6 independent pools of roots derived from three plants). Germinated plants were grown on a 1% agar plate for 4 d without any nutrients. Then, they were transferred to a new agar plate containing 0 or 5 mM KNO_3 and were grown for 6 h before sampling. RT-qPCR data were normalized to WT non-treated conditions. The expression of *LjUBQ* was used as the reference. Scatterplots show individual biological replicates as dots. Bars indicate mean values. Different letters indicate statistically significant differences ($P < 0.0001$, two-way ANOVA followed by multiple comparisons).

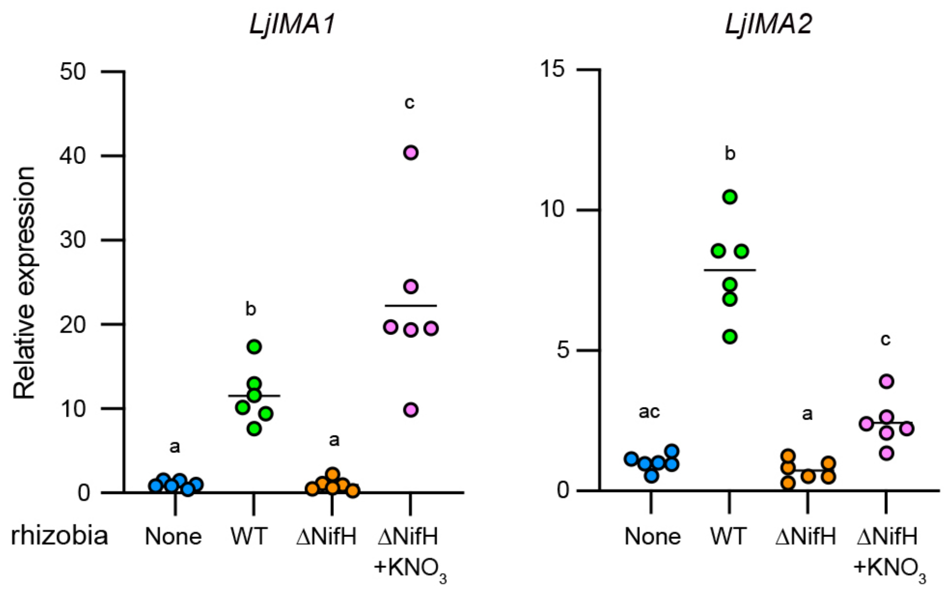

Supplementary Fig. 11 Effects of nitrate treatment on *LjIMA1/2* expression in RNS.

RT-qPCR analysis of *LjIMA1/2* genes in the 11 dai shoot in non-inoculated, WT and ΔNifH rhizobia-inoculated conditions. Whole shoots of *L. japonicus* WT plants were collected for RT-qPCR analysis (n = 6 independent pools of shoots derived from three plants). For nitrate treatment, the plants were inoculated with ΔNifH rhizobia and grown for 9 d without nitrogen nutrients, then they were treated with 10 mM KNO_3 and grown for 2 d before sampling. RT-qPCR data were normalized to WT non-inoculated conditions. The expression of *LjUBQ* was used as the reference. Scatterplots show individual biological replicates as dots. Bars indicate mean values. Different letters indicate statistically significant differences ($P < 0.05$, two-way ANOVA followed by multiple comparisons).

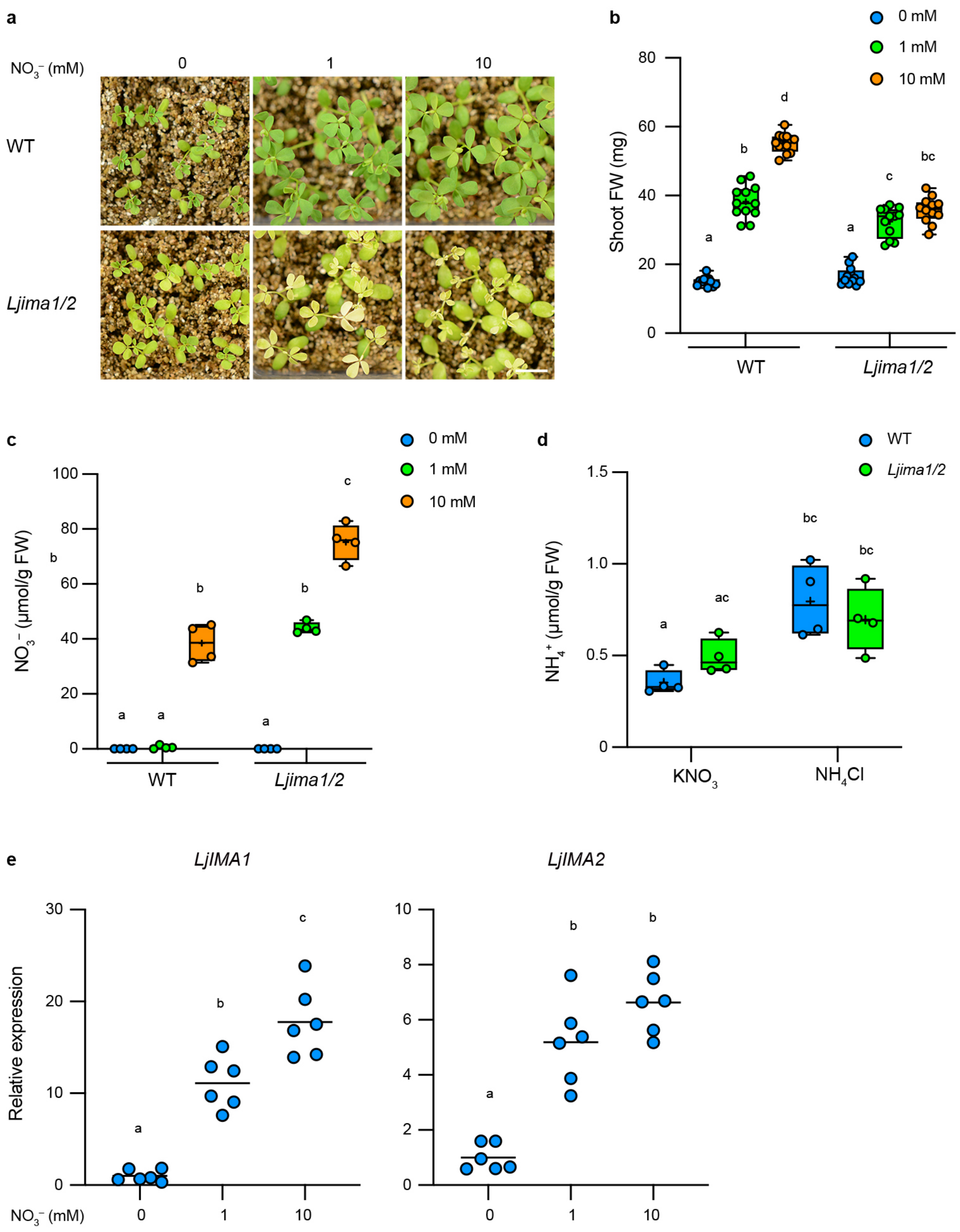

Supplementary Fig. 12 Effects of nitrate treatment on plant growth.

a-d. Shoot phenotypes, shoot FW (n = 12 plants), shoot nitrate (n = 4 independent pools of shoot derived from three plants) and ammonium (n = 4 independent pools of shoot derived from three plants) contents of WT and *Ljima1/2* grown in different nitrate concentrations for 17 d after germination in the absence of rhizobia. **e.** RT-qPCR analysis of *LjIMA1/2* expression in WT in response to different concentrations of nitrate. WT shoots were collected 2 d after treatment with 1 or 10 mM KNO_3 in the absence of rhizobia (n = 6 independent pools of shoots derived from three plants). RT-qPCR data were normalized to non-treated conditions. The expression of *LjUBQ* was used as the reference. Scale bar, 1 cm. Centerlines in the boxplots show the medians, and box limits indicate the 25th and 75th percentile. The whiskers go down to the smallest value and up to the largest. Scatterplots show individual biological replicates as dots. In **e.**, bars indicate mean values. In **b.c.d.e.**, different letters indicate statistically significant differences ($P < 0.01$, one- or two-way ANOVA followed by multiple comparisons).

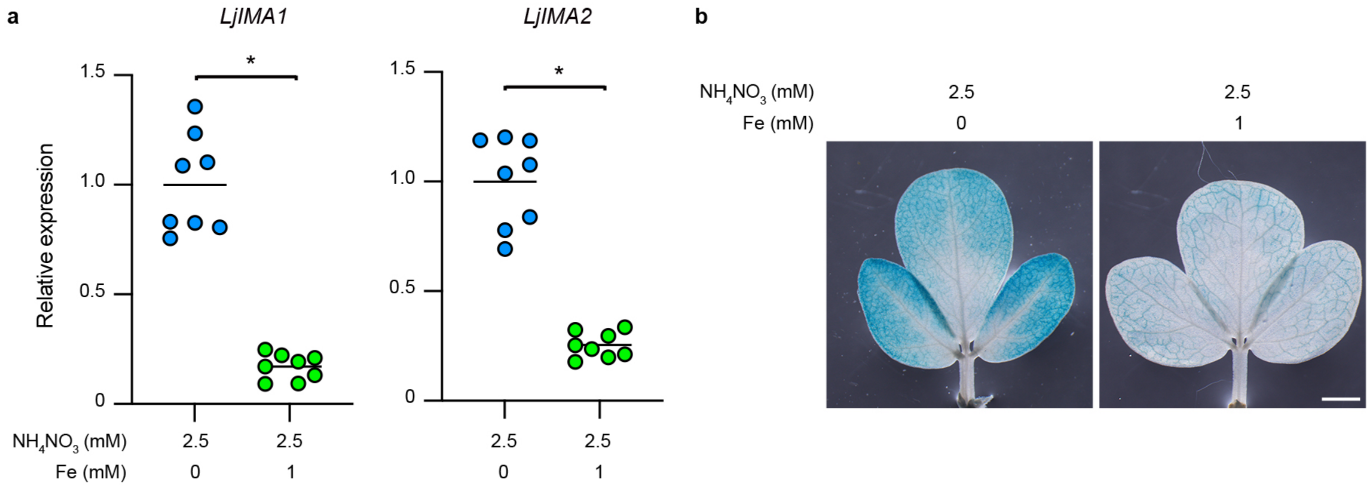

Supplementary Fig. 13 *LjIMA1/2* expression in response to nitrogen-Fe balance.

a. RT-qPCR analysis of *LjIMA1/2* expression in WT. WT shoots were collected 2 d after treatment with 2.5 mM NH_4NO_3 with (1 mM) or without Fe in the absence of rhizobia (n = 8 independent pools of shoots derived from three plants). RT-qPCR data were normalized to –Fe conditions. The expression of *LjUBQ* was used as the reference. Scatterplots show individual biological replicates as dots. In **a.**, bars indicate mean values. Asterisks indicate a statistically significant difference ($P < 0.0001$, by a two-sided Welch's *t* test). **b.** GUS activities of $LjIMA1_{pro}$:*GUS* plants in different nitrogen-Fe conditions. Two independent experiments were conducted to obtain similar results. Scale bar, 1 mm.

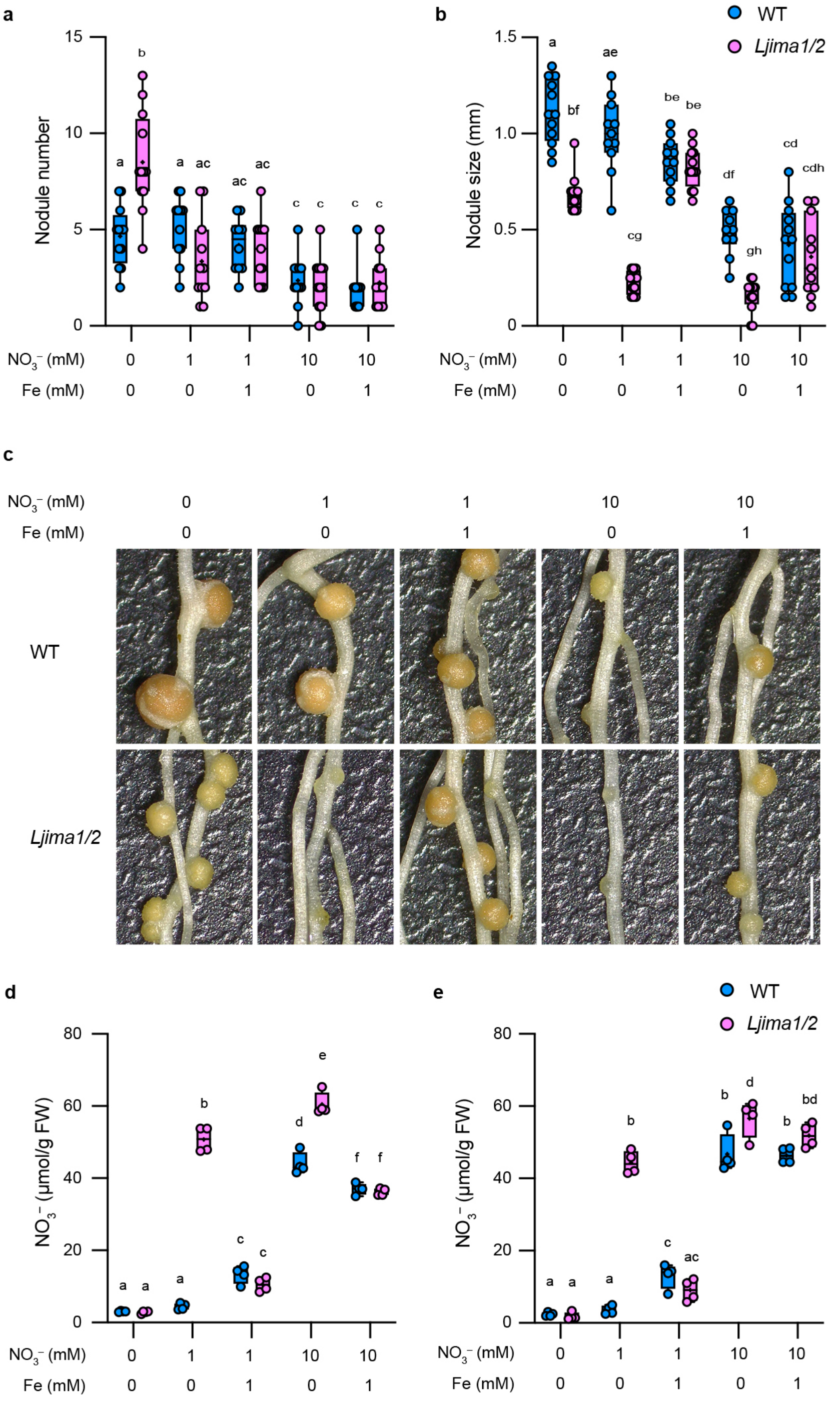

Supplementary Fig. 14 Nitrate inhibition of nodulation in response to nitrogen-Fe balance.

a-c. Nodulation phenotypes of WT and *Ljima1/2* 14 dai in different nitrate and Fe concentrations. **a.** Total nodule number (n = 10-12 plants). **b.** Maximum nodule diameter of nodules on the root of each plant (n = 10-12 plants). **d.e.** nitrate contents of WT and *Ljima1/2* 14 dai in different nitrate and Fe concentrations (n = 4 independent pools of shoots or roots derived from 2-3 plants). **d.** shoots. **e.** roots. Scale bar, 2 mm. Centerlines in the boxplots show the medians, and box limits indicate the 25th and 75th percentile. The whiskers go down to the smallest value and up to the largest. Scatterplots show individual biological replicates as dots. Different letters indicate statistically significant differences ($P < 0.05$, two-way ANOVA followed by multiple comparisons).

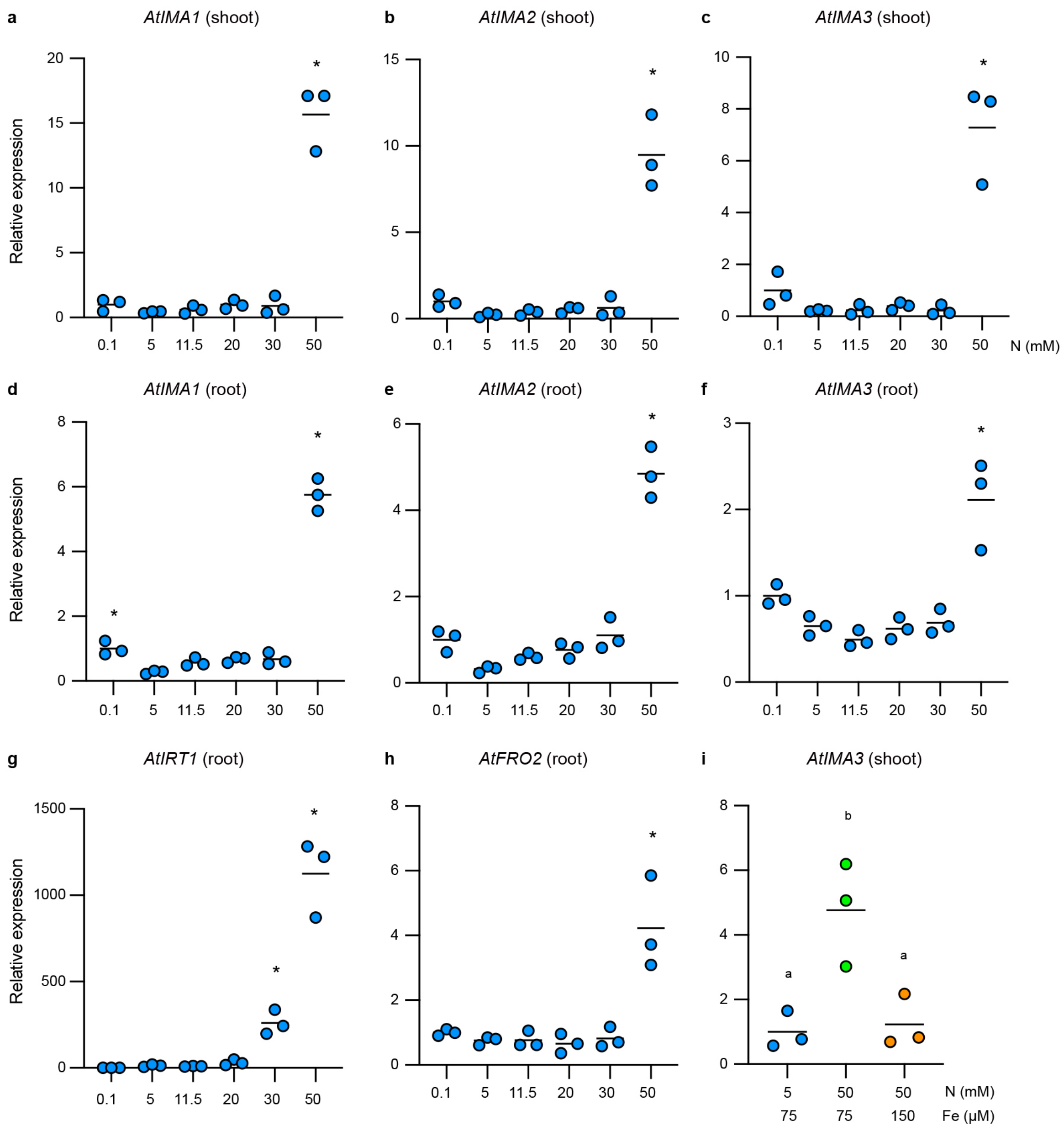

Supplementary Fig. 15 *AtIMAs* expression in response to different concentrations of nitrogen nutrients.

a-h. RT-qPCR analysis of *AtIMA1/2/3*, *AtIRT1* and *AtFRO2* expression in *Arabidopsis thaliana* WT (Col-0) in response to different concentrations of nitrogen nutrients. Plants were grown on agar plates with 1/2 MS medium containing 5 mM nitrogen nutrients for 7 d, and then they were transplanted to new plates with different concentrations of nitrogen nutrients, including KNO_3 and NH_4Cl. 3 d later, shoots and roots were collected (n = 3 independent pools of shoot or root derived from three plants). **i.** RT-qPCR analysis of shoot *AtIMA3* expression by different nitrogen-Fe ratios. Prior to sampling, plants were grown in each condition with different nitrogen-Fe ratios for 7 d (n = 3 independent pools of shoot derived from three plants). The expression of *At18SrRNA* was used as the reference. Scatterplots show individual biological replicates as dots. Bars indicate mean values. In **a-h.**, asterisks indicate a statistically significant difference compared with 5 mM N ($P < 0.05$, one-way ANOVA followed by multiple comparisons). In **i.**, different letters indicate statistically significant differences ($P < 0.05$, one-way ANOVA followed by multiple comparisons).
