# Supplementary material for: IMA peptides regulate root nodulation and nitrogen homeostasis by providing iron according to internal nitrogen status
Source: Nat Commun. 2024 Jan 29;15:733. doi: 10.1038/s41467-024-44865-4 (PMC10825120; doi:10.1038/s41467-024-44865-4)
Supplement: Supplementary file 3 — Description of Additional Supplementary Files [file 41467_2024_44865_MOESM3_ESM.pdf]

Description of Additional Supplementary Files:

Supplementary Dataset 1: DEG in response to internal nitrogen status during RNS.

Supplementary Dataset 2: Enriched GO terms of up-regulated genes by LjIMA1/2

Supplementary Dataset 3: Primers used in this study.

Supplementary Dataset 4: Gene IDs described in this study.
